# The impact of chronotype on prosocial behavior

**DOI:** 10.1371/journal.pone.0216309

**Published:** 2019-04-30

**Authors:** Natalie L. Solomon, Jamie M. Zeitzer

**Affiliations:** 1 PGSP Stanford Psy.D. Consortium, Palo Alto University, Palo Alto, California, United States of America; 2 Department of Psychiatry and Behavioral Sciences, Stanford University, Stanford, California, United States of America; 3 Mental Illness Research Education and Clinical Center, VA Palo Alto Health Care System, Palo Alto, California, United States of America; Middlesex University, UNITED KINGDOM

## Abstract

**Introduction:**

Chronotype (morningness/eveningness) is associated with preference for the timing of many types of behavior, most notably sleep. Chronotype is also associated with differences in the timing of various physiologic events as well as aspects of personality. One aspect linked to personality, prosocial behavior, has not been studied before in the context of chronotype. There are many variables contributing to who, when, and why one human might help another and some of these factors appear fixed, while some change over time or with the environment. It was our intent to examine prosocial behavior in the context of chronotype and environment.

**Methods:**

Randomly selected adults (N = 100, ages 18–72) were approached in a public space and asked to participate in a study. If the participants consented (n = 81), they completed the reduced Morning-Eveningness Questionnaire and the Stanford Sleepiness Scale, then prosocial behavior was assessed.

**Results/Conclusions:**

We found that people exhibited greater prosocial behavior when they were studied further from their preferred time of day. This did not appear to be associated with subjective sleepiness or other environmental variables, such as ambient illumination. This suggests the importance of appreciating the differentiation between the same individual’s prosocial behavior at different times of day. Future studies should aim at replicating this result in larger samples and across other measures of prosocial behavior.

## Introduction

Individual differences in the time at which people prefer to do particular behaviors, most notably sleep, are referred to as chronotype. In essence, chronotype describes whether someone is a “morning” person or an “evening” person. While many (50%) individuals identify somewhere between the extremes of chronotype, around 30% of individuals identify as morning type and 20% identify as evening type [1,2]. An individual’s chronotype is likely created by an interaction between the endogenous circadian pacemaker and its responses to light [3,4] and can be modulated by factors such as age [2,5,6] and life circumstance (e.g., needing to get to work early over years may shift preference towards earlier hours). There are a variety of physiologic events that vary by chronotype (e.g., timing of melatonin [7], core temperature [8], and cortisol [9]), as well behaviors that vary by chronotype (e.g., cognition, mood, susceptibility to stress and personality traits) [10,11]. A meta-analysis examining the association between chronotype and personality, as described by the Big Five Personality Model [12], found that conscientiousness is the personality dimension that relates most to morningness. Agreeableness is also related to morningness, although to a lesser degree, and openness to experience, extraversion and neuroticism, contribute a very small degree [12].

Another variable linked to both agreeableness and conscientiousness, prosocial behavior, has received little attention in terms of its potential modulation by chronotype. Prosocial behavior, or an action that is done for the benefit of another human or society as a whole, is regulated by both situational and dispositional variables [13]. The study of situational determinants of prosocial behavior was the focus of most early investigation. Among the situational variables that could influence prosocial behavior are setting (rural settings eliciting more prosocial behavior than urban settings) [14,15], other behaviors (e.g., cell phone use) [16], amount of sunlight [17], and weather [18]. Noise has also been found to be negatively correlated with prosocial behavior, with high noise levels interfering more with verbal help than with physical help [19]. Opportunities in which the situation is viewed as uncontrollable, such as a medical emergency, are likely to evoke more prosocial behavior [20], while the presence of bystanders reduces prosocial behavior [21]. More recently, however, there has been increasing interest in examining how dispositional (trait) variables relate to prosocial behavior. Agreeableness and conscientiousness are the personality traits most correlated with prosocial behavior [22–24]. Other variables associated with prosocial behavior include sex [25] and age [26,27].

As both chronotype and prosocial behavior are linked to agreeableness, conscientiousness, and other aspects of personality, we wanted to explore whether chronotype is linked to prosocial behavior. One previous study examining adolescents found morningness to be correlated positively with prosocial behavior, and negatively with behavioral problems [28]. We specifically hypothesized that individuals would be more likely to engage in prosocial behavior if asked to do so when closer to their preferred time of day. We secondarily hypothesized that sleepiness, a common occurrence in many adults that can be associated with chronotype [29] and impacts many aspects personality [30], would be negatively associated with prosocial behavior.

## Materials and methods

Participants (N = 100) were approached at the Mountain View Caltrain Station in Mountain View, California. This location was chosen because many people waiting at the station may have some extra time and may not be immediately headed somewhere. Participants were approached when a train was not scheduled to depart from the platform within the next eight minutes. The same researcher (NS) approached all individuals. The researcher approached every third person on the platform to reduce the likelihood that the researcher was biasing their choice of participant. If the next participant was within earshot of the last participant, the researcher would move on to the next person on the platform who was out of earshot of the last participant.

To control for the effects of socializing and peer influence, only individuals standing alone were sampled. Individuals with others standing nearby were approached while individuals clearly traveling with another were not. Children (individuals who appeared to be less than 17 years old), people on crutches, people with heavy packages or others who might not be fully capable of filling out the questionnaires were excluded.

Data were collected at two time points: morning (between the hours of 5:00 am and 10:00 am) and evening (between the hours of 5:00 pm and 10:00 pm). These morning and evening hours were chosen in order to study the behavior of both morning and evening type people in both the morning and evening. Once a participant had been identified, the researcher estimated the participant’s age, gender, ethnicity, and recorded if they were using a cellphone. The researcher also recorded if the participant was standing alone or if others were present.

When participants were approached, they were told the following: “Hi, I am from the PGSP-Stanford Consortium and we're conducting a survey of sleep. Would you be willing to answer a few questions to contribute to our research? Your participation is completely voluntary.” Individuals who agreed to participate were given a one-page survey that included three demographic questions and six sleep related questions.

When the participant finished the survey, the researcher thanked them and employed a sidewalk interview method [14,18,31,32] by saying the following script, which was adapted from the sociology department of the University of Minnesota [18]: “We are also conducting a second study related to sleep. Although the survey is 80 questions long, you do not have to answer all of the questions. How many questions would you be willing to answer to help me?” If the participant asked how long the questions on the second survey would be, the researcher responded that they were similar in length to the questions on the previous survey. The number of questions the respondent agreed to answer was used as a measure of time-giving prosocial behavior (i.e., 0–80). After responding, the participants were debriefed about the nature of the study (there was no additional set of questions). The use of this deception was explicitly discussed with the Stanford University Institutional Review Board, who approved the study prior to any data collection. The study follows the principles laid out in the Declaration of Helsinki. The entire interaction between the researcher and each participant took approximately two-three minutes.

### Questionnaires

Immediate levels of subjective sleepiness was assessed through completion of the one-item Stanford Sleepiness Scale (SSS) [33]. The scale has been widely validated for adult populations and is extensively used in the literature to assess current feelings of sleepiness. Chronotype was determined using the reduced Morning-Eveningness Questionnaire (rMEQ) [34].The rMEQ can be used to divide individuals into “morning”, “evening”, and “neither” chronotypes.

### Environmental factors

Temperature and humidity readings (AcuRite Pro Accuracy Temperature and Humidity Monitor, Model #01083M, Lake Geneva WI; range: -20-70°C, 1–99% relative humidity) were taken at the beginning of each hour in which participants were approached. A sound level reader (Decibel Sound Meter Pro, v.2.9.1, iPhone 7 application) was used for 15 seconds at the beginning of each hour in order to measure the average level of noise in the immediate area; this number was used to describe the noise level in the subsequent hour. Smartphone-based assessment of sound, while not as good as a professional-grade monitor, yields reasonably accurate data for the purposes of assessing general levels of ambient noise [35]. A digital photometer (Spectracine Professional IV A, Spectra Cine, Burbank CA) was also used at the start of each hour in order to measure light. Light measurements were taken in duplicate at 1.8 m in both the downward angle of gaze as well as horizontal angle of gaze.

### Statistical analyses

X^2^ and Fisher tests were performed using an online platform (http://vassarstats.net/). All other statistical analyses were performed using IBM SPSS Statistics software (version 23.0, IBM Corp., Armonk NY) and Microsoft Excel (v. 16.0.4639.1000, Microsoft Corp., Redmond WA). Data are presented as mean ± SD.

## Results

**Fig 1:**
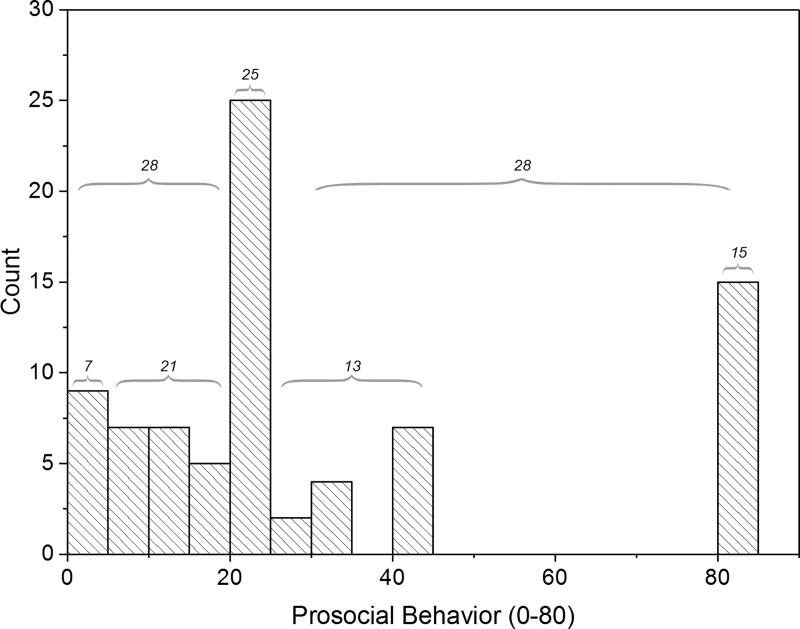
Histogram of prosocial behavior. We, *post hoc*, categorized participants into those who displayed low prosocial behavior (scores 0–19), moderate prosocial behavior (scores of 20), and elevated prosocial behavior (scores 21–80).

Individuals were studied during nine separate collection points between June 2017 and March 2018. Of the 100 individuals approached, 81 agreed to participate in this study (Table 1). Across all data collection periods, temperature ranged 8.6–31°C (21±6.0°C), relative humidity ranged 24–63% (46±12%), illuminance ranged 10–776 lux (259±191 lux, downward angle of gaze) and 5.7–903 lux (335±213, horizontal angle of gaze), and ambient sound ranged 49.8–67.65 dB (59.5±3.94 dB). At no point during the data collection was there rain or other inclement weather. Individuals were relatively alert, with a median SSS score of 2 and 88% scoring between 1 and 3 (between fully alert and not quite fully alert) (*n* = 74). rMEQ scores ranged between 8 and 24 with a median score of 17 (*n* = 74). These rMEQ scores could be converted into categories of: 4 definite morning types, 22 moderate morning types, 43 neither morning nor evening types, and 5 moderate evening types. Prosocial behavior did not have a normal distribution, rather it appeared categorical (Fig 1). As such, in addition to using prosocial behavior as a continuous variable, we converted it into three evenly distributed categorical variables: low (0–19, n = 28), moderate (20, n = 25), and high (21–80, n = 28).

**Table 1:** Sample demographics (n = 81).

	N (%)
**Gender**	
Male	47 (58%)
Female	30 (37%)
Did not disclose	4 (5%)
**Ethnicity**	
Caucasian	40 (50%)
African American	1 (1%)
Asian	25 (31%)
Latino	6 (7%)
Other	5 (6%)
Did not disclose	4 (5%)
**Age**	
18–25 years	13 (16%)
26–35 years	30 (37%)
36–55 years	25 (31%)
56–75 years	8 (10%)
Did not disclose	5 (6%)

**Fig 2:**
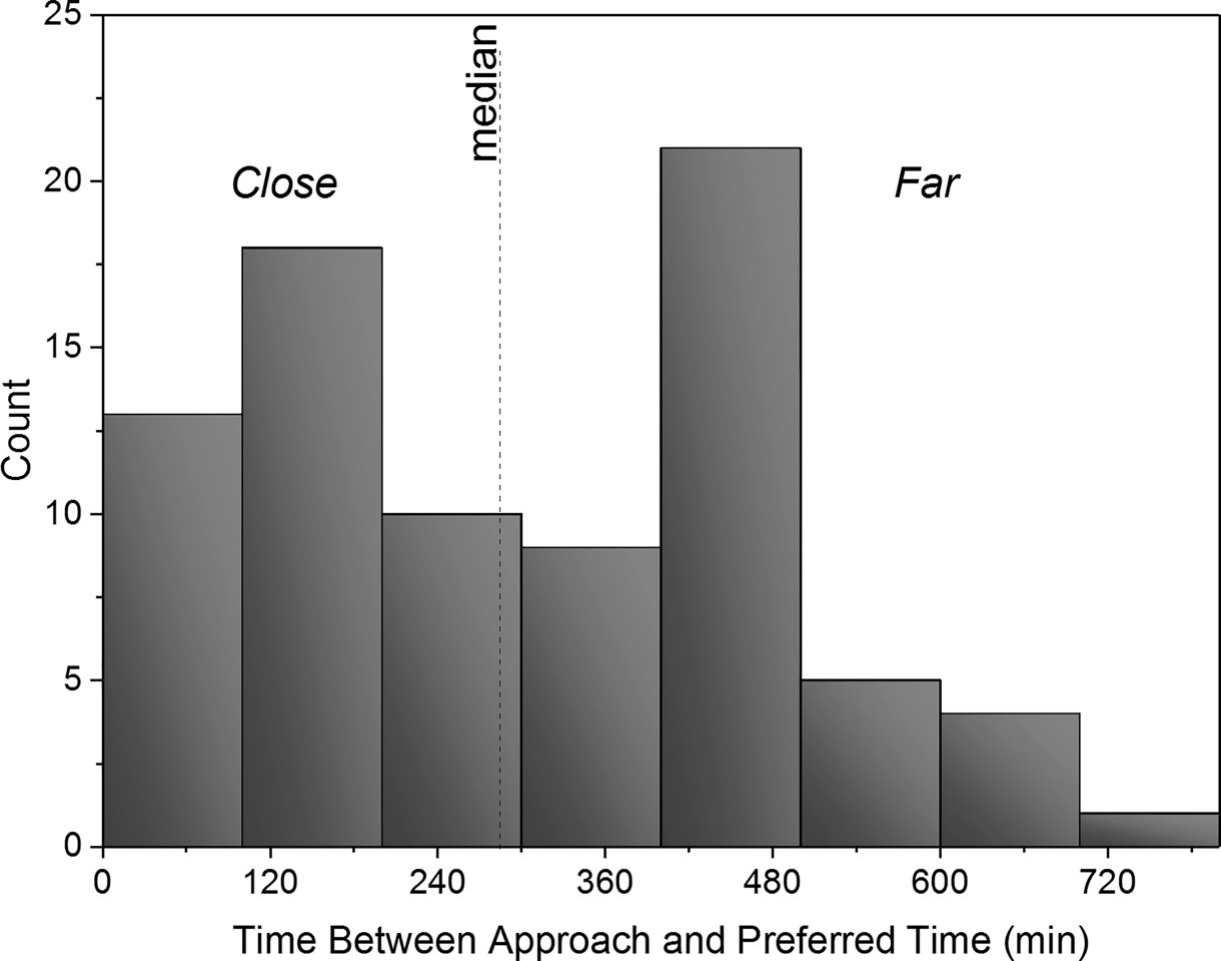
Histogram of minutes between time of participation preferred time. A median split was used to separate individuals who were studied “close” to or “far” away from their preferred time of day as determined by chronotype questionnaire.

To determine whether individuals were “near to” or “far from” their preferred time of day (e.g., a person who identified their feeling best time to be in the morning and who is being studied in the evening would be considered “far from their preferred time of day” and categorized as “far”), we first parsed participants into two groups: Close (people asked to engage in prosocial behavior close to their preferred time of day) and Far (people asked to engage in prosocial behavior far from their preferred time of day). How close someone was to their preferred time of day was calculated by calculating the difference between the time the participant was approached and the time they identified as their “feeling best” peak on the rMEQ. The median of these values was used to split the participants into Close and Far (Fig 2). When examined in this way, we observed a relationship between prosocial behavior and whether a person was asked to help close to or far from their preferred time of day (X^2^ (4, N = 81) = 10.42, p<0.05), such that people were more likely to exhibit prosocial behavior when further away from their preferred time of day (e.g., a person who identified their feeling best time to be in the evening, asked to help in the morning). As chronotypes being studied out of their preferred time of day may exhibit increased sleepiness, we also examined the relationship between sleepiness scores and prosocial behavior, but found no relationship (Spearman rank-order correlation; *r*_*s*_(74) = -0.12, *p* = 0.31). Independently, there was no association between prosocial behavior and absolute rMEQ score (Spearman rank-order correlation; *r*_*s*_(81) = 0.05, *p* = 0.65). We also did not observe and association between prosocial behavior and ambient noise, temperature, or humidity (Spearman rank-order correlations; |*r*_*s*_|’s(81) < 0.03, *p’s* > 0.79).

When perspective participants were approached, a similar percentage of people were using their cellphone in those who accepted participation (58%) as those declined (75%) (Fisher exact test, p = 0.27). We did not observe a difference in prosocial behavior based on prior cellphone use (X^2^ (4, N = 81) = 0.75, p = 0.945). While cellphone use was not associated with prosocial behavior, we also examined whether angle of gaze (i.e., looking up or down) was associated with prosocial behavior. When perspective participants were approached, a similar percentage of people were looking down in those who accepted participation (67%) as those who declined (84%) (Fisher exact test, p = 0.221). While prosocial behavior was not associated with angle of gaze when prosocial behavior was examined categorically (X^2^ (4, N = 81) = 8.83, p = 0.066), it was associated when prosocial behavior was examined as a continuous variable (t-test, t = -2.09, df = 79, p < 0.05), such that a downward angle of gaze prior to being approached was associated with lower subsequent prosocial behavior. As direction of gaze could influence the amount of light reaching the participant, and there is a robust relationship between the intensity of ambient lighting and mood [36], we also examined whether ambient light levels (measured in the downward angle of gaze for those looking down and horizontal angle of gaze in those not looking down) were associated with prosocial behavior. There was, however, no relationship between light intensity in the direction of gaze and prosocial behavior considered as a continuous variable (Spearman rank-order correlation, *r*_*s*_(79) = 0.05, *p* = 0.64) or categorical variable (ANOVA, df = 4, F = 0.95, p = 0.44).

## Discussion

Research on morningness/eveningness has gained much attention recently. While most early research focused on biological correlates, such as the relative timing of melatonin, cortisol, and body temperature [37], more recently, psychological correlates (e.g., mental health [10] or susceptibility to stress [11]), have been investigated. Moreover, the concept of morningness-eveningness has found its way into personality research [38]. In adolescents, morningness has been associated with engagement in prosocial behavior [28]. Further findings on a relationship between morningness and prosocial behavior, however, are scarce.

In this study, we found that people engage in more time-giving prosocial behavior when they are further from their preferred time of day. For instance, someone who prefers the evening time may be more likely to engage in time-giving prosocial behavior in the morning. This relationship was unlikely to be mediated by any association between chronotype and sleepiness, as we did not observe a direct correlation between subjective sleepiness and prosocial behavior.

While we did not observe a correlation between subjective sleepiness and prosocial behavior, distance from preferred time of the day may be related to tiredness rather than sleepiness. Fatigue or tiredness may be linked to one’s use of intuitive strategies versus deliberative strategies. Research suggests that cooperation in one-time interactions is not automatic, but appears only at later stages of reasoning [39]. Interestingly, it has also been found that promoting intuition versus deliberation has no effect on cooperative behavior [40]. However, depletion has been linked to dishonesty [41] and reduced prosocial behavior [42]. Future research could examine chronotype, tiredness and prosocial behavior.

We found a relationship between a downward angle of gaze and reduced prosocial behavior and that this is unlikely to be mediated by reduced ambient illumination or cellphone use. In order to better understand why individuals who are asked to engage in prosocial behavior close to their optimal “feel-good” time are less likely to be helpful, more research is needed. A possible explanation is that during an individual’s “feel-good” time, they may be more protective of their optimal ‘thinking time’ (especially in the circumstances in which the behavior was probed). This would be consistent with our observation that individuals with their heads pointed downward, perhaps an indication of contemplative thinking or listening to an internal dialogue [43], are less likely to engage in prosocial behavior. Future research is necessary to test these hypotheses.

A cost–reward analysis of helping assumes an economic view of prosocial behavior, stating that people are motivated to maximize their rewards and to minimize their costs [44]. In a potential helping situation, a person analyzes the circumstances, weighs the probable costs and rewards of alternative courses of action, and then arrives at a decision that will result in the best outcome [13]. Therefore, helping is more likely to occur when the rewards for helping outweigh the costs. However, costs and rewards are subjectively determined and there is considerable individual variation in responses. Our hypothesis that one’s time may be valued differently and influence subsequent time-giving prosocial behavior at varied times of day fits into this perspective.

In many psychological studies including the present study, prosocial behavior is examined in the context of short-term encounters with strangers. This methodology excludes other types of prosocial behaviors, for example those prescribed by the female gender role, as they are displayed primarily in long-term, close relationships. Future research could examine how different dimensions of prosocial behavior may map onto the construct of chronotype. Further, we had a limited number (*n =* 5) of participants who were evening types. As such, we were unable to examine whether chronotype independent of time of day is related to prosocial behavior. Larger studies that specifically recruit based on this characteristic would be needed to understand this question.

Previous research examined the influence of environmental variables on prosocial behavior [18]. Given the moderate climate in the Northern California area in which we approached individuals, we were unable to examine whether swings in weather or large changes in sunlight exposure that occur seasonally were able to influence prosocial behavior. We were also unable to replicate the association between either noise, temperature, or humidity, and prosocial behavior, though the lack of a large dynamic range in these environmental variables may have contributed to our findings.

Our findings, including that one may engage in more time-giving prosocial behavior further from their preferred time of day, are useful for organizations and individuals. In organizations, some aspects of scheduling could be informed by the likelihood of time-giving prosocial behavior. For individuals, one’s chronotype may influence whether one engages in prosocial behavior at a given time. This is important as it influences how one may view themselves and is likely to impact self-concept, self-esteem and future behavior. The capacity to perform some behaviors at different times of day allows people to set their schedules to capitalize on times of peak performance in different domains. More research will be necessary to examine the determinants of the relationship between chronotype and prosocial behavior and how this could be used to optimize general well-being.
